# Clinical Case Study Investigating a Longevity Daily Serum for Japanese Women With Facial Skin Aging

**DOI:** 10.1111/jocd.70206

**Published:** 2025-05-07

**Authors:** Mayumi Nomoto, Morgann Young, Tatiana Kononov, Alisar Zahr

**Affiliations:** ^1^ Nomoto Mayumi Clinic Ginza Tokyo Japan; ^2^ Nomoto Mayumi Skin Care Clinic Niigata Japan; ^3^ Revision Skincare Irving Texas USA

To the Editor,

Mitochondrial health is important for skin health and well‐aging [1, 2]. When skin is exposed to damaging intrinsic and extrinsic factors, mitochondria are negatively impacted, leading to dysfunction, reduced energy (adenosine triphosphate), and impaired membrane potential [2]. At this stage, skin may be clinically characterized by dehydration, lack of visual firmness, and a dull appearance [2].

We have developed a longevity daily serum (LDS) [2] to optimize and boost skin's energy with mitochondria‐targeting ingredients. The LDS is an oil‐in‐water emulsion formulated with a unique sunflower sprout extract (SSE) [3] containing beneficial secondary metabolites, such as omega 3‐fatty acids, acetyl‐L‐carnitine, and adenosine triphosphate (ATP); an antioxidant blend with tetrahexyldecyl (THD) ascorbate (Vitamin C); as well as microbiome technology (postbiotic blend: *Bacillus* and *Saccharomyces* ferment), and dermal epidermal junction (DEJ) targeting technology (Iris Florentina root extract) [2]. Pre‐clinical studies have demonstrated that the LDS increases skin's cellular energy, improves mitochondrial membrane potential, and protects skin from extrinsic oxidative stress [4]. Clinically, the LDS improved skin hydration, dullness, sagging, and overall photodamage in American females over 7 days [2] and 12 weeks [5]. These studies led to the current case study involving healthy Japanese women (Data S1). The primary objective was to assess the efficacy and tolerability of the LDS in improving skin radiance, firmness, and laxity. It was hypothesized that the LDS would be effective and well tolerated.

Five subjects 45–52 years old (average 48 years) with Fitzpatrick Skin Type III (2 subjects) and IV (3 subjects) completed the study. Due to the small sample size, clinical grading results were not statistically significant; however, compelling insights emerged. Across the five subjects, the LDS‐treated side saw a greater trend in improvement at Week 12 (Figure 1). Compared to a placebo serum (PS), the LDS provided greater improvements in global facial overall appearance and radiance as well as global facial and jawline skin sagging. Specifically, jawline skin sagging improved by Week 4 on the LDS‐ versus the PS‐treated side. Additionally, 60% of subjects improved at Week 12 on the LDS side with a 40% median improvement (*p* = 0.375) versus a 20% median improvement (*p* = 0.375) on the PS side. This data, although directional, indicates the effectiveness of the LDS.

**FIGURE 1:**
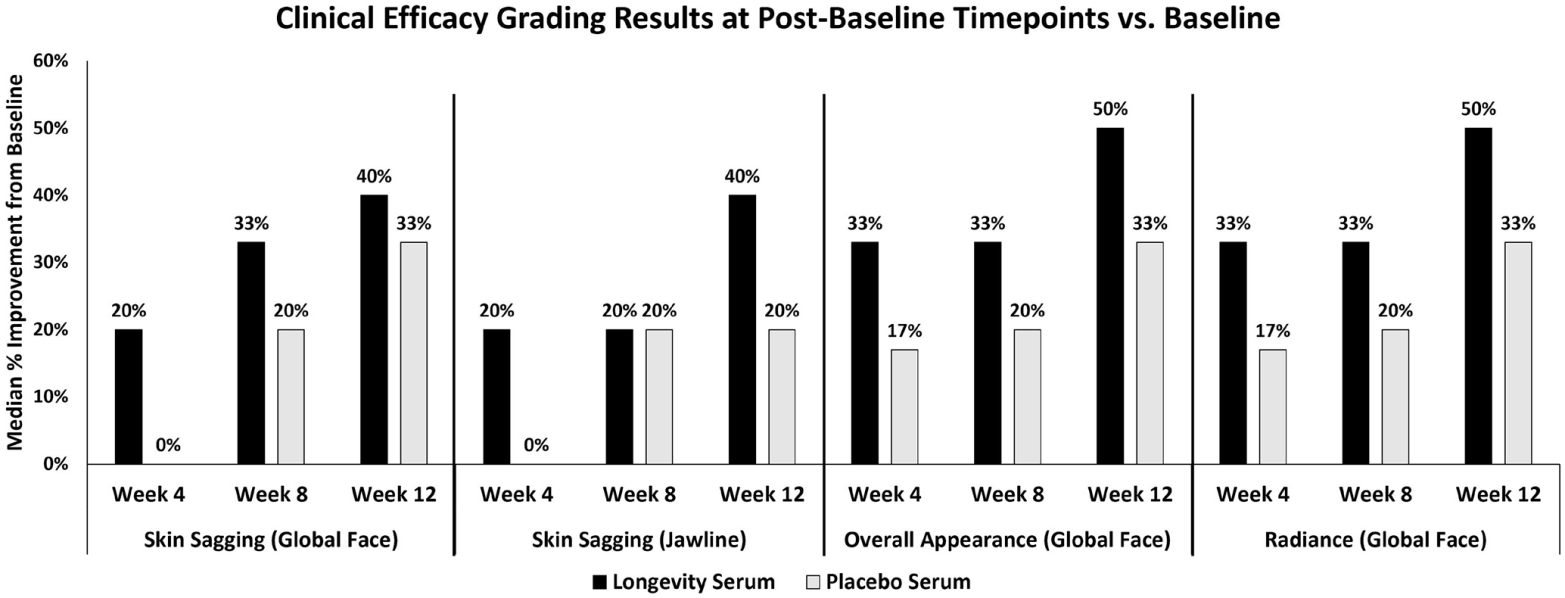
Clinical efficacy grading results at post‐baseline timepoints. Data represent median percent improvement from baseline for skin sagging, overall appearance, and radiance. Median percent improvement is based on the change in median scores across all subjects at Weeks 4, 8, and 12 compared to baseline.

Skin sagging improvement on the LDS‐treated side was captured by QuantifiCare LifeViz 3D photography (Figures 2 and 3). Colors moving towards red represent increasing length and lift compared to baseline. Arrows pointing upward and downward indicate improved and worsened skin sagging, respectively. Both subjects demonstrated a reduction in skin sagging on the LDS‐treated side, evident by the upward arrows, whereas the PS side has downward arrows, representing skin sagging from baseline to Week 12.

**FIGURE 2:**
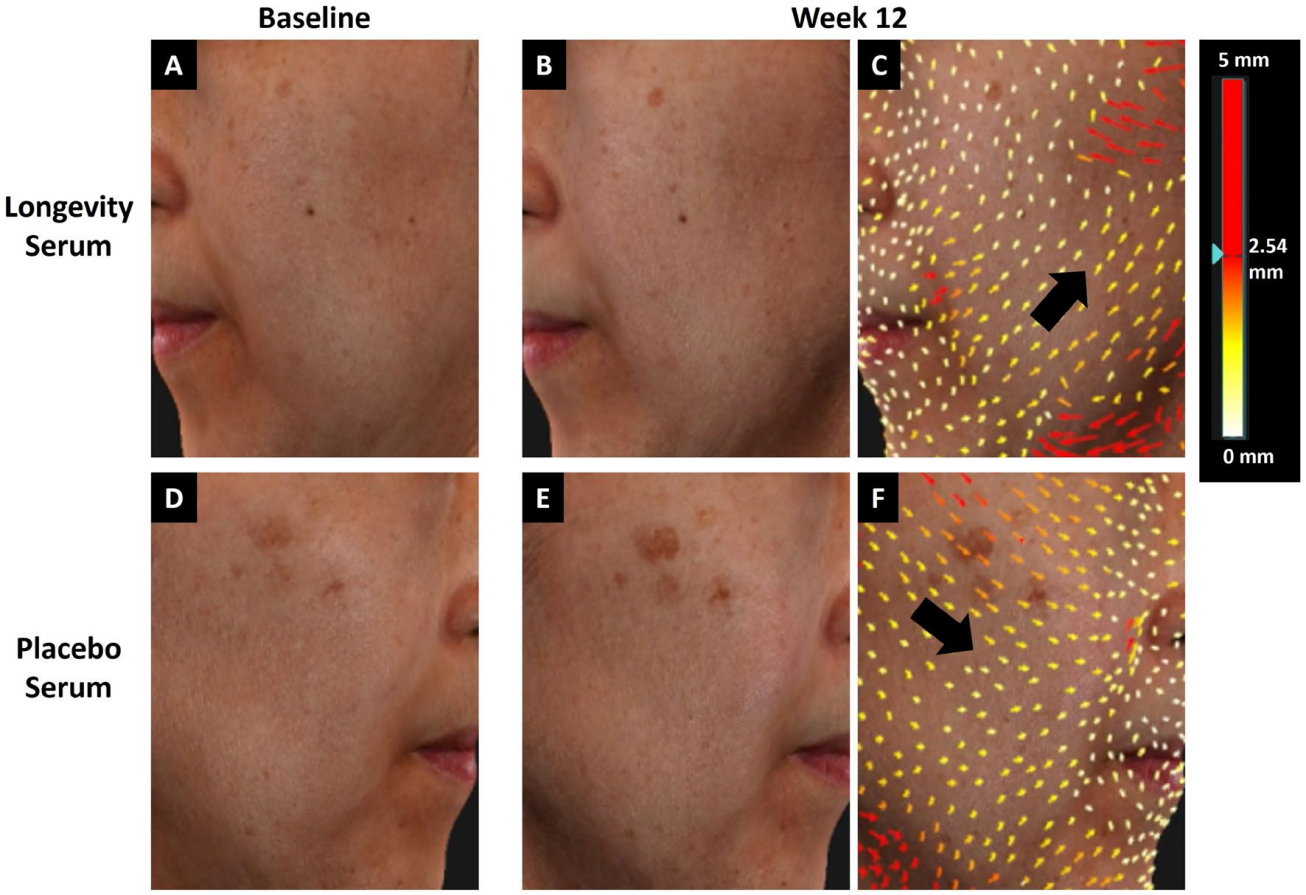
QuantifiCare LifeViz 3D lifting photographs with color scale ranging from white (0 mm lift) to red (max 5 mm lift). The subject is 49 years, Fitzpatrick Skin Type IV. (A–C) Longevity serum–treated side at baseline and Week 12. (D–F) Placebo serum–treated side at baseline and Week 12.

**FIGURE 3:**
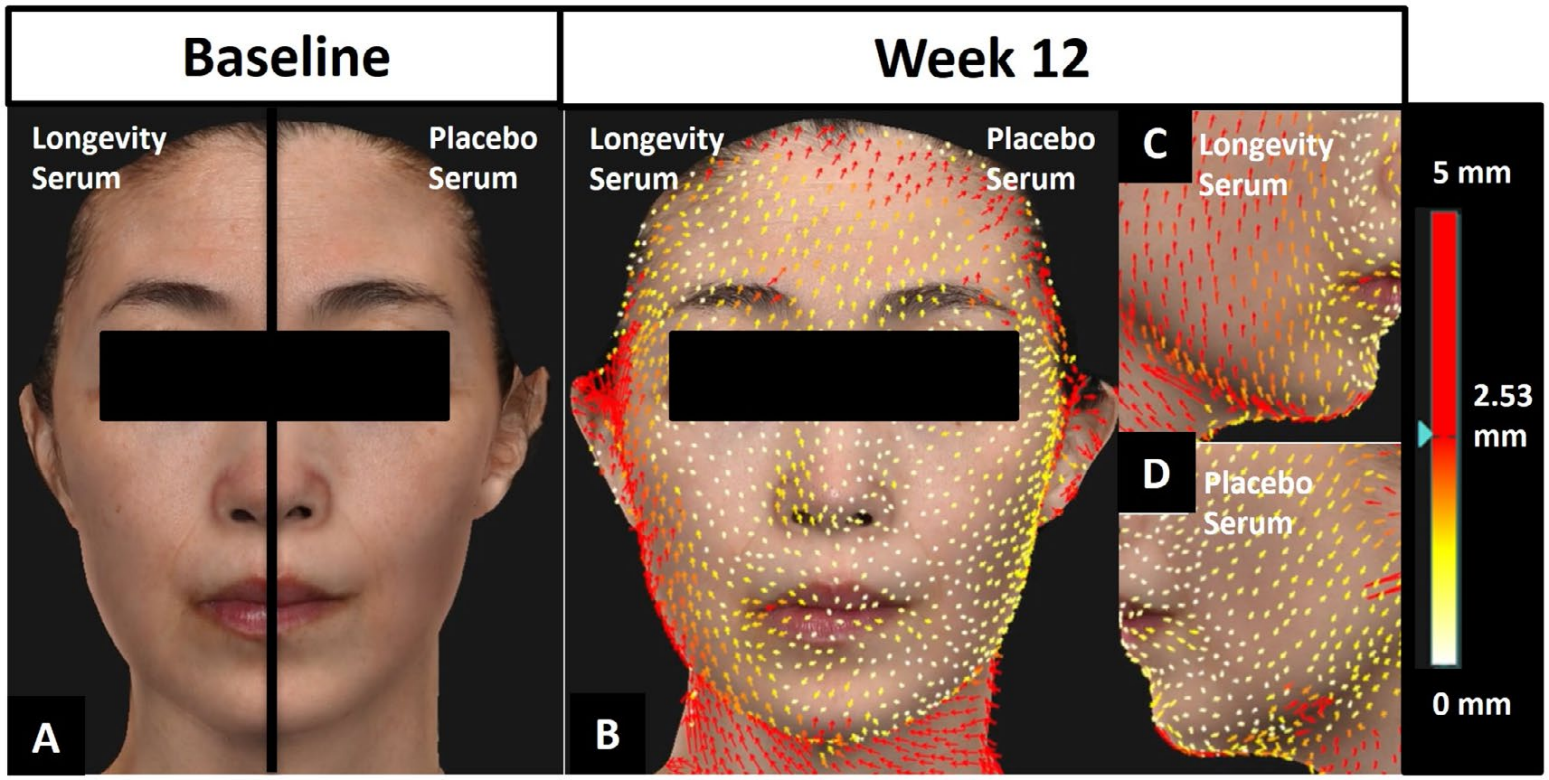
Quantificare LifeViz 3D lifting photographs with color scale ranging from white (0 mm lift) to red (max 5 mm lift). The subject is 52 years old, Fitzpatrick Skin Type III. (A) Baseline and (B–D) Week 12.

At Week 12, self‐assessment questionnaire results showed that 60% (LDS) versus 40% (PS) of subjects favorably agreed that “My complexion is glowing,” and “My skin looks healthy.” [6] Sixty percent (LDS) versus 0% (PS) of subjects favorably agreed that “My skin appears firm,” and 100% (LDS) versus 40% (PS) of subjects favorably agreed that “My skin tone appears more even” [6].

The objectives of this case study were achieved with clinical improvements observed over 12 weeks. As demonstrated by efficacy grading and 3D photography, the LDS established a trend in improved skin radiance, firmness, and laxity (sagging) versus the PS‐treated side. The LDS and PS were well‐tolerated, and there were no reported adverse events. The limitation of this study was the small sample size. Additionally, future studies with a larger population and longer study duration will be performed.

These positive findings could be attributed to the mitochondria‐targeting ingredients within the LDS, including the unique SSE, which contains multiple secondary metabolites like omega 3‐fatty acids, acetyl‐L‐carnitine, and ATP [2]. With the efficacy and safety demonstrated in Japanese women, the LDS can be recommended to Japanese women looking for skincare treatments targeting skin radiance, firmness, and laxity. Formulated to energize skin cells and promote mitochondrial health, the LDS is an effective treatment for patients looking to optimize skin energy and longevity.

## Conflicts of Interest

Dr. Mayumi Nomoto, MD, PhD has performed clinical trials and consulting for a variety of organizations and serves in multiple leadership capacities. Dr. Mayumi Nomoto, MD, PhD was the principal investigator and clinical grader for this clinical trial. Morgann Young, MS and Dr. Alisar Zahr, PhD analyzed the images and statistical results as well as drafted the manuscript. At the time of submission, Morgann Young, MS was an employee of Revision Skincare and now is an employee of Galderma. Alisar Zahr, PhD is an employee of Revision Skincare. Tatiana Kononov, BS, MBA is a consultant for Revision Skincare and assisted in the draft of the manuscript. The serum evaluated in the clinical study is a marketed Revision Skincare product.

## Supporting information


Data S1.

## Data Availability

The data that support the findings of this study are available from the corresponding author upon reasonable request.
