# Supplementary material for: Clinical Case Study Investigating a Longevity Daily Serum for Japanese Women With Facial Skin Aging
Source: J Cosmet Dermatol. 2025 May 7;24(5):e70206. doi: 10.1111/jocd.70206 (PMC12056886; doi:10.1111/jocd.70206)
Supplement: Supplementary file 1 — Data S1. [file JOCD-24-e70206-s001.docx]

Clinical Case Study Investigating a Longevity Daily Serum for

Japanese Women with Facial Skin Aging

**Supplementary Material**

**Materials and Methods**

**Study Design**

A 12-week single-center, randomized, double-blinded, split-face, placebo-controlled case study was conducted to evaluate the efficacy and tolerability of the longevity daily serum (LDS) in five healthy Japanese female subjects 45-70 years of age with Fitzpatrick Skin Types II-IV and moderate to severe facial skin sagging (score of 4-9 on the modified Griffiths scale; 0 = best and 9 = worst skin condition).

In addition, subjects needed to have not used topical facial products containing actives such as vitamin C, retinoids, and alpha/beta/poly-hydroxy acids within 4 weeks prior to baseline and be willing to replace all topical facial products with the provided test products during the study. Subjects who were nursing, pregnant, or planning a pregnancy during the study were not eligible.

All subjects were provided with an explanation of the study and allowed to ask any questions before agreeing to participate. An informed consent form was signed by each subject.

Subjects completed a 3-day washout using a gentle foaming cleanser, toner, and basic moisturizer on the global face, jawline, and neck morning and evening. A basic sunscreen SPF 30 PA+++ was also applied in the morning on the same areas and reapplied as needed. Starting at baseline and per split-face randomization, subjects applied the active (LDS) and placebo serum (PS) on the global face (including upper eyelid), jawline, and neck after toner morning and evening. Products were to be applied to the right and left side using the corresponding hand. Subjects used a basic cream if they experienced skin dryness (note subjects needed the basic cream during the study to alleviate dryness resulting from the winter season in Tokyo, Japan). While the skincare regimen was multifaceted, the objective was to maintain alignment with the fundamental skincare needs of this demographic.

**Evaluations**

Clinical efficacy and tolerability grading, clinical photography, and self-assessment questionnaires were completed at baseline and weeks 4, 8, and 12. For all assessments, each side of the face was evaluated.

Clinical efficacy grading was performed by a board-certified dermatologist according to a 10-point modified Griffiths scale, where a score of 0 indicates best skin condition and 9 indicates worst skin condition; a decrease in score represents an improvement. Multiple parameters were evaluated, including global facial overall appearance and radiance. Skin sagging of the global face and jawline was also graded based on tactile evaluation.

Tolerability grading was completed by the dermatologist (erythema, edema, scaling/peeling) and each subject (burning/stinging, itching, tightness/dryness) according to a 4-point scale, where 0 is none and 3 is severe. A decrease in score indicates an improvement.

QuantifiCare LifeViz® (QuantifiCare, Biot, France) three-dimensional photographs were captured to visualize skin movement, or an improvement in skin sagging, at week 12.

In addition, subjects completed a self-assessment questionnaire on product efficacy and aesthetics according to a 5-point scale, where 1 is Completely Disagree and 5 is Completely Agree.

**Statistical Analysis**

The Wilcoxon Signed Rank Test was used to analyze median scores from clinical and tolerability grading. A 90% confidence interval was utilized with significance set at **p* < 0.10. Top box analysis was performed for the self-assessment questionnaire; ratings of 4 (Slightly Agree) and 5 (Completely Agreed) were considered favorable agreement.
